# Time from US Food and Drug Administration approval to publication of data for cancer drugs: a comparison of first and subsequent approvals

**DOI:** 10.1038/s41408-017-0008-9

**Published:** 2017-11-30

**Authors:** Austin Lammers, Ruibin Wang, Jeremy Cetnar, Vinay Prasad

**Affiliations:** 1 Division of Hematology Oncology, Knight Cancer Institute, Oregon Health & Science University, 3181 SW Sam Jackson Park Road, Portland, OR 97239 USA; 2 Department of Epidemiology, Johns Hopkins Bloomberg School of Public Health, Baltimore, MD USA; 3 Department of Public Health and Preventive Medicine, Oregon Health & Science University, Portland, OR 97239 USA; 4 Center for Health Care Ethics, Oregon Health & Science University, Portland, OR 97239 USA

In 1993, the Omnibus Budget Reconciliation Act required the Centers for Medicare and Medicaid Services (CMS) to pay for off-label cancer drugs if supported by one of the five compendia, which over time has come to include that of the National Comprehensive Cancer Network (NCCN)^1^. Estimates suggest that 30% of cancer drug use in the United States (US) is off-label, amounting to annual costs of nearly 5 billion dollars in 2010^2^. Typically, these cancer drugs are initially approved for one indication, but once supported by a compendium; they may be used for other indications and be covered.

The quality of evidence cited in the compendia for off-label cancer drugs may not rise to the same level of evidence as FDA-approved drugs^2^. For this reason there is a theoretical incentive for companies to achieve FDA approval for one indication, and seek subsequent compendia inclusion for alternative uses. This may be preferable to seeking FDA approval for each and every indication. Since reimbursement for subsequent indications does not require formal FDA approval, there may be less incentive to pursue it.

Given that formal US Food and Drug Administration approval has not been required for the reimbursement for off-label uses, we hypothesized that there may be less incentive for companies to see seek subsequent approvals, a higher regulatory hurdle than compendia inclusion. We therefore sought to compare the time from study publication in the peer review literature to approval between first and subsequent approvals in order to assess for this difference. If there were less incentive to seek subsequent approvals, the average time from publication (used for compendia inclusion) to approval would be longer.

To do this, we ascertained all US FDA hematology and oncology anti-cancer drug approvals from the FDA website: http://www.fda.gov/Drugs/InformationOnDrugs/ApprovedDrugs/ucm279174.htm from 2010 to 2014. We excluded approvals that were solely changes in drug formulation; conversion of accelerated to traditional approval, as well as rare indications infrequently encountered in clinical oncology (e.g., everolimus for tuberous sclerosis with subependymal giant cell astrocytoma).

Marketing authorization was noted to be the first or subsequent approval. For each approval, we reviewed drug label citations referenced for efficacy data. In order to ascertain the date of publication, we searched Google Scholar and Medline to identify the peer review publications that reported the efficacy data referenced in the drug approval, using combination of search terms, involving the disease, indication and drug name.

Additionally, we searched clinicaltrials.gov for the date if/when efficacy data was posted. Of note, the Food and Drug Administration Amendments Act (FDAAA) mandates reporting of results to that website^3^. We wanted to know the time in which this was typically performed with respect to the date of approval. Statistical analysis was performed with STATA v.12.0 (College Station, TX). Descriptive statistical analysis was performed. Waterfall plots were made using R.

From 2010 to 2014, the US FDA approved 83 marketing indications for anti-cancer drugs, and 75 were eligible our analysis. Table 1 shows the characteristics of the 75 approvals, with 27 (36%) approvals based on response rate, 27 (36%) based on progression-free survival, and 21 (28%) based on overall survival. Forty authorizations (53.3%) were first approvals and 35 (46.6%) were subsequent.

**Table 1:** Characteristics of cancer drugs that received US FDA approval from 2010 to 2014

Characteristics	
Number of drugs approved	75
Published based on RR, *N* (%)	27 (36.0)
Published based on PFS, *N* (%)	27 (36.0)
Published based on OS, *N* (%)	21 (28.0)
Accelerated approvals, *N* (%)	23 (30.7)
Single arm studies, *N* (%)	14 (56.0)
Subsequent approvals, *N* (%)	35 (46.7)
With results posted on Clinicaltrials.gov, *N* (%)	65 (86.7)
With articles from US FDA’s Oncology Drug Products, *N* (%)	40 (53.3)
Range of days from publication to FDA approval	−1193, 361
Days from publication to FDA approval, median (IQR)	−33 (−182, 86)
Range of days from posting on Clinicaltrials.gov to FDA approval, min, max	−460, 1761
Days from posting on Clinicaltrials.gov to FDA approval, median (IQR)	96 (59, 438)

**Fig. 1:**
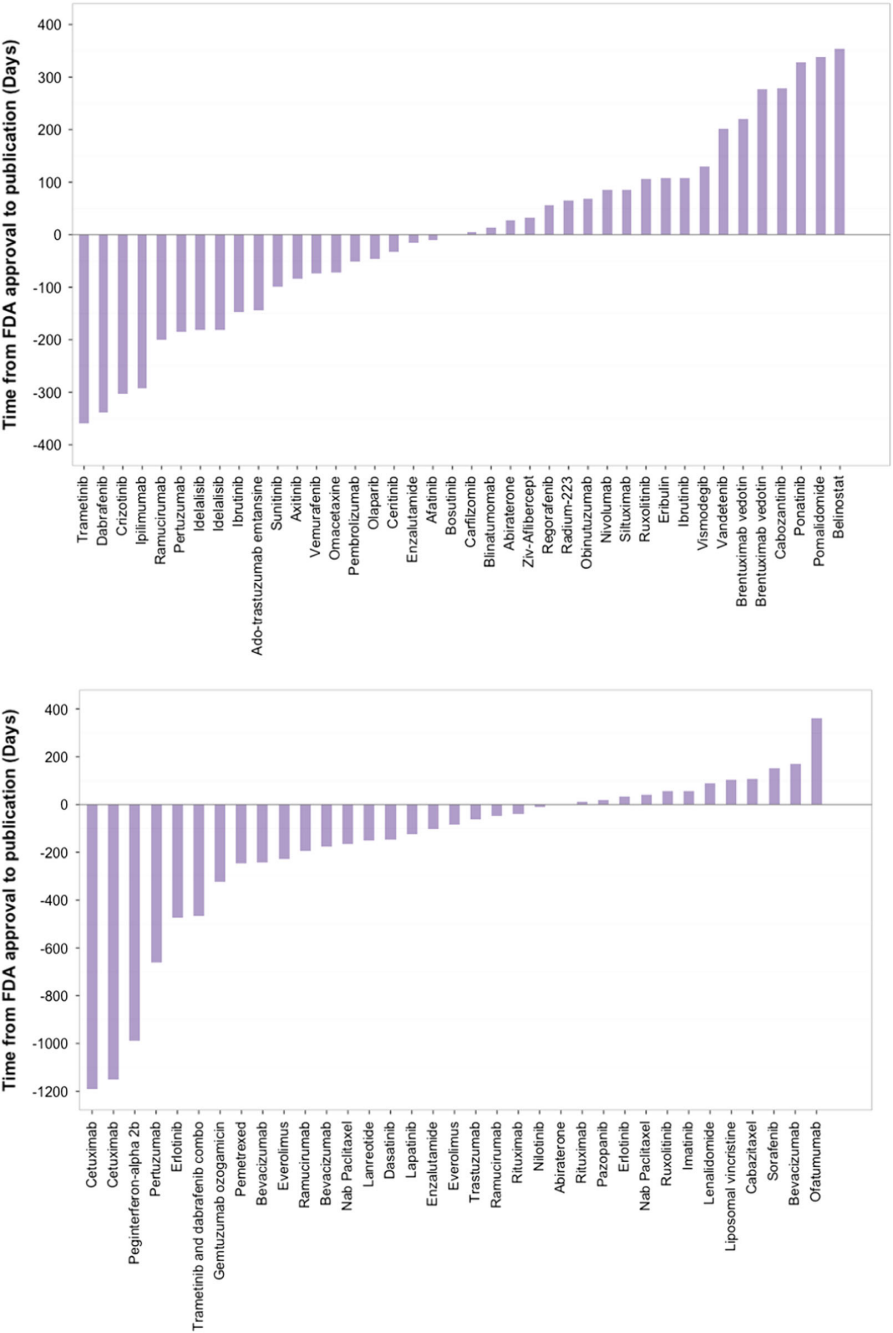
**a**, **b** Time from US Food and Drug Administration approval to publication for first (top panel) and subsequent approvals (bottom panel) Negative numbers indicate publications that precede approval, while positive numbers indicate publications that came after approval. Drug names appear more than once among first approvals when two approvals were granted on the same day. Time from US FDA approval to Publication of Pivotal Study Results (first approvals). Time from US FDA approval to Publication of Pivotal Study Results (subsequent approvals)

Figure 1 depicts the time from publication to approval for first (top panel) and subsequent (bottom panel) marketing authorizations. Publication preceded approval by a longer period for subsequent approvals than first approvals (median (inter quartile range) days from approval to publication −84 days (−242, 40) for subsequent approvals and 2.5 days (−121.5, 107) for first approvals, *p* = 0.034).

Twenty-seven (36.0%) approvals did not have efficacy data published in the peer review literature within 30 days of approval. Only two of these approvals posted results on Clinicaltrials.gov in this time, and none of these approvals had the FDA publish efficacy data within this time.

We found that the time from pivotal trial publication to FDA approval was significantly longer for subsequent than first cancer drug approvals. (Fig. 1). One likely driver is that the incentive to seek formal, subsequent drug approval is limited, as Medicare is obliged to pay for any drug recommended by one of several compendia. It is unlikely due to longer times for processing FDA applications, as data from the FDA reveals those times are shorter^4^.

Arguably, the bar for compendia inclusion is lower than that of FDA approval. For instance, just 8% of NCCN recommendations are based on category I evidence^5^, and some run counter to FDA decisions. Bevacizumab was revoked by the US FDA for use in metastatic breast cancer, yet remains in the NCCN guidelines. Thus, current incentives may foster a culture where drug companies seek approval for any indication, and subsequently perform weaker studies to gain inclusion for several other purposes, occasionally seeking a subsequent approval.

These issues are only becoming more complex. The 21st Century Cures Act provides regulatory pathways for the use of real-world data to leverage further approval^6, 7^. Thus, the incentive to even conduct subsequent clinical trials, let alone publish them may be further reduced. The generation of credible evidence to guide cancer care remains an undisputed good. Whether and to what extent real world, retrospective observational data can fulfill that remains unknown^6^.

Our investigation reveals a sizable percentage (36.0%) of cancer drug approvals do not have published efficacy data within 30 days of approval. We found efficacy data are rarely available from other sources. Timely publication of clinical trial data remains vital for optimal clinical decision-making.

The off-label use of cancer drugs is, to some degree, necessary, particularly for older cancer drugs that lack patent protection. Yet, current policies are now utilized by newer, costly drugs, broadening market share, and may discourage sponsors from seeking formal approval, based on robust studies, for these off-label uses.
